# A Gammaherpesviral Internal Repeat Contributes to Latency Amplification

**DOI:** 10.1371/journal.pone.0000733

**Published:** 2007-08-15

**Authors:** Nagendra N. Thakur, Susanne El-Gogo, Beatrix Steer, Klaus Freimüller, Andreas Waha, Heiko Adler

**Affiliations:** 1 Institute of Molecular Immunology, Clinical Cooperation Group Hematopoietic Cell Transplantation, GSF National Research Center for Environment and Health, Munich, Germany; 2 Department of Medicine III, Ludwig Maximilians University of Munich, Munich, Germany; 3 Institute of Virology, Technical University of Munich, Munich, Germany; 4 Institute of Neuropathology, University of Bonn, Bonn, Germany; Institut Pasteur Korea, Republic of Korea

## Abstract

**Background:**

Gammaherpesviruses cause important infections of humans, in particular in immunocompromised patients. The genomes of gammaherpesviruses contain variable numbers of internal repeats whose precise role for *in vivo* pathogenesis is not well understood.

**Methodology/Principal Findings:**

We used infection of laboratory mice with murine gammaherpesvirus 68 (MHV-68) to explore the biological role of the 40 bp internal repeat of MHV-68. We constructed several mutant viruses partially or completely lacking this repeat. Both *in vitro* and *in vivo*, the loss of the repeat did not substantially affect lytic replication of the mutant viruses. However, the extent of splenomegaly, which is associated with the establishment of latency, and the number of *ex vivo* reactivating and genome positive splenocytes were reduced. Since the 40 bp repeat is part of the hypothetical open reading frame (ORF) M6, it might function as part of M6 or as an independent structure. To differentiate between these two possibilities, we constructed an N-terminal M6STOP mutant, leaving the repeat structure intact but rendering ORF M6 unfunctional. Disruption of ORF M6 did neither affect lytic nor latent infection. In contrast to the situation in lytically infected NIH3T3 cells, the expression of the latency-associated genes K3 and ORF72 was reduced in the latently infected murine B cell line Ag8 in the absence of the 40 bp repeat.

**Conclusions/Significance:**

These data suggest that the 40 bp repeat contributes to latency amplification and might be involved in the regulation of viral gene expression.

## Introduction

Gammaherpesviruses like Epstein-Barr virus (EBV) and Kaposi's sarcoma-associated herpesvirus (KSHV) are lymphotropic viruses which establish lifelong infections in their hosts and are associated with cellular transformation and the development of malignancies, particularly in immunosuppressed individuals such as transplant recipients or AIDS patients [1]–[3]. The genomes of human gammaherpesviruses contain variable numbers of internal repeats [4]–[8]. Repeats are also found in coding regions and it is known that in some cases the repeats are indeed translated into protein [4]. One example is the glycine-alanine repeat in the EBV nuclear antigen 1 (EBNA 1) [9]–[11]. It has been described earlier that there is frequently no third position variation (degeneration) in the repeats, implying that they might have functions apart from coding for a protein which may prevent these repeat sequences from drifting [4]. The DR1 and DR2 repeats in the KSHV latent viral protein Kaposin B are required for the cytokine mRNA stabilization function of Kaposin B [12], [13]. Furthermore, it has been observed that the number of repeat units varies among naturally occuring virus isolates [6], [14]. However, despite numerous *in vitro* studies [8], [15]–[19], the precise role of such internal repeats for *in vivo* pathogenesis is not well understood.

Until recently, pathogenesis studies with human gammaherpesviruses have been hampered by the species specificity and the difficulties and expense inherent in analyzing pathogenesis in primate models. Infection of laboratory mice with murine gammaherpesvirus-68 (MHV-68), which was originally isolated from the bank vole (*Clethrionomys glareolus*) [20], provides a small animal model for addressing basic aspects of gammaherpesvirus pathogenesis and immunity [21]–[23]. The presence of two different internal repeats in the genome of MHV-68 offers the possibility to address the role of internal repeats in gammaherpesvirus pathogenesis. The 100 bp internal repeat is located to the right side of the genome (nucleotide positions 98981–101170) and consists of approximately 21 copies of 100 bp repeat units, whereas the 40 bp internal repeat is present at the left side of the genome (nucleotide positions 26778–28191) and consists of approximately 36 copies of 40 bp repeat units [24].

Here, we investigated the biological role of the 40 bp internal repeat by infecting mice with mutant viruses partially or completely lacking this repeat. The 40 bp internal repeat appears to be largely dispensable for lytic replication both *in vitro* and *in vivo*, however, it plays a role during amplification of latency. This phenotype seems to be dependent on the number of the 40 bp repeat units present in the genome and is not associated with expression of the hypothetical ORF M6. The expression of the latency-associated genes K3 and ORF72 was reduced during latent infection of the murine B cell line Ag8, but not during lytic infection of NIH3T3 cells, with a mutant virus lacking the 40 bp repeat. These data suggest that the 40 bp repeat contributes to latency amplification and seems to be involved in the regulation of viral gene expression.

## Materials and Methods

### Cell culture and virus stocks

Ag8 cells were maintained in RPMI 1640 medium (Invitrogen, Germany) and NIH3T3 cells in DMEM (Invitrogen, Germany), both supplemented with 10% fetal calf serum, penicillin (100 U/ml), streptomycin (100 µg/ml) and 2 mM L-glutamine. BHK-21 cells were maintained in Glasgow's modified Eagle's medium (GIBCO) supplemented with 5% fetal calf serum, 5% tryptose-phosphate broth, penicillin (100 U/ml), streptomycin (100 µg/ml) and 2 mM L-glutamine. Virus stocks were prepared using BHK-21 cells as described previously [25]. Viral titers were determined by plaque assay on BHK-21 cells as described [25].

### Construction of recombinant BAC plasmids

Mutant viruses were constructed using MHV-68 cloned as a BAC as described [25]. A deletion mutant (Delta 40 bp mutant), completely lacking the 40 bp internal repeat of MHV-68, was constructed by ET cloning [26], [27] as described [25]. Briefly, the 40 bp repeat was first replaced by a tetracycline resistance gene flanked by FRT (FLP recognition target) sites, resulting in the deletion of nucleotides 26778 to 28191. The tetracycline resistance gene was then removed by FLP-mediated recombination, leaving behind only a small residual insert consisting of a FRT site and some vector sequence including a new *EcoR*I restriction site. A revertant virus was generated by replacing the 40 bp mutant fragment with a wild type genomic fragment (nucleotide positions 24918–30834) by a two step replacement procedure as described [25], [28]. Briefly, a 5.916 kbp *Sma*I fragment of MHV-68 (nucleotide positions 24918–30834) was cloned into the shuttle plasmid pST76K-SR which was then used for the two-step replacement procedure. As described previously [25], BAC clones of MHV-68 may loose a certain number of the 40 bp internal repeat units due to recombination between the direct repeats. Two of such clones were selected for this study, the 40 bp low mutant which had lost a considerable number of the 40 bp repeat units and the 40 bp moderate mutant which had lost some of the repeat units. The number of the remaining repeat units in the 40 bp low mutant is 15 as determined by sequencing. The corresponding number in the 40 bp moderate mutant is approximately 20 to 25 repeat units as estimated from restriction enzyme analysis. An additional mutant (M6STOP) was generated by targeting the N-terminus of ORF M6 using the two-step replacement procedure. For this purpose, a 5.234 kbp fragment of MHV-68 (nucleotide positions 24918–30152) was used for the insertion of a 16 bp STOP-linker containing a *Hpa*I-site and stop codons in all possible frames [29] into the *Hind*III site at nucleotide position 26711. The mutated fragment was cloned into the shuttle plasmid pST76K-SR and used for the two-step replacement procedure. As described, the 40 bp repeat has the propensity to be unstable in *E. coli* [24], [25]. As a result, some of the 40 bp repeat units were lost during the generation of the M6STOP mutant. All recombinant BAC plasmids were analyzed by restriction enzyme analysis and/or sequencing.

### Reconstitution of recombinant viruses

Recombinant viruses were reconstituted as described [25]. After reconstitution of infectious viruses, the BAC vector sequences were removed by infection of Cre-expressing rat embryonic fibroblasts as described [25]. DNA of all recombinant viruses was isolated from infected cells by the method described by Hirt [30] and analyzed by restriction enzyme digestion and Southern blot as described [25]. A probe (spanning nucleotides 25889 to 26711) was prepared by PCR, labelled with Digoxygenin using the DIG-labeling Kit from Roche (Mannheim, Germany) and used for Southern blot analysis according to the instructions of the manufacturer.

### In vitro growth analysis

To analyze the *in vitro* growth of the recombinant viruses, NIH3T3 cells were infected with 0.1 PFU per cell. Cells together with supernatants were harvested at different time points after infection and stored at –80°C. The amount of infectious virus in the cultures was determined after two rounds of freezing and thawing by plaque assay on BHK-21 cells as described [25].

### In vivo infections

All animal experiments were approved by the appropriate local authorities. C57BL/6 mice were obtained from Charles River Laboratories, Sulzfeld, Germany. CD8^−/−^ mice on a C57BL/6 background and the corresponding wildtype C57BL/6 mice were purchased from Jackson Laboratories (Bar Harbor, Maine). Female mice were infected intranasally (i.n.) at 8–10 weeks of age with 5×10^4^ PFU in 40 µl of PBS. Prior to i.n. infection, mice were anesthetized with ketamine/xylazine. After infection, mice were housed in individually ventilated cages (ivc) for the time of the experiment. At the indicated time points after infection, mice were sacrificed and organs were obtained for further analysis. Lungs were harvested and frozen at −80°C. Virus titers in the lungs were determined by plaque assay after tissue homogenization and two rounds of freezing and thawing as described [31]. Spleens were harvested and the weight and cell number were determined. Single cell suspensions were used for the *ex vivo* reactivation assay as described [31].

### Measurement of latent viral load by quantitative real time PCR

Latent viral load in the spleens of infected mice was quantified by real-time PCR using the ABI 7300 Real Time PCR System (Applied Biosystems, Foster City, CA). Amplification was performed with Taqman universal PCR master mix and universal cycling conditions (Applied Biosystems, Foster City, Calif.). Quantification of viral DNA copy number was made by amplification of a 70 bp region of the MHV-68 gB gene using primers and probes as described by others [32]. A standard curve was generated using known amounts of a plasmid containing the *Hind*III-N fragment of MHV-68 (containing the gB gene). DNA was extracted from spleen cells using the QIAAMP DNA MINI KIT (Qiagen, Hilden, Germany) and quantified by UV spectrophotometry. One hundred nanograms of DNA were used per reaction. The murine ribosomal protein L8 (rpl8) was amplified in parallel and used to normalize for input DNA between samples. The primer and probe sequences for L8 were as follows: Forward: 5′-CATCCCTTTGGAGGTGGTA-3′; Reverse: 5′-CATCTCTTCGGATGGTGGA-3′ and Probe: 5′-ACCACCAGCACATTGGCAAACC-3′. A standard curve for rpl8 was constructed by serial dilution of a plasmid containing rpl8 (RZPD clone IRAVp968B01123D6) (RZPD, Berlin, Germany). The data are presented as viral genome copy numbers relative to the copy number of L8.

### RT-PCR and real-time PCR analysis of viral transcripts

Total RNA from infected or uninfected cells (NIH3T3 or Ag8) was isolated using the TRI- reagent (SIGMA, Munich, Germany). DNAse-treated RNA (2 µg) was reverse transcribed using the 1^st^ strand cDNA Synthesis Kit for RT-PCR (AMV)^+^ from Roche (Mannheim, Germany) in a 20 µl reaction. A reverse transcriptase control (without reverse transcriptase) was included in the experiments. The primer sequences were as follows [33]: ORF K3: 5′-GTCGCGATCGCCTCATCAATG-3′ and 5′-GAGAGTTCTGTTGGATCTGC-3′, ORF 72: 5′-TGTGATTAGCACTGGGCGTTTC-3′ and 5′-TATCGCAGCGAAAGAGAACACG-3′, ORF 73: 5′-TAGATCCAGGTGATCCTGTGGC-3′ and 5′-CCGCATAATCCATCTGATCCAT-3′. The conditions for the PCR were as follows: initial denaturation at 94°C for 3 min, followed by 30 cycles of 94°C denaturation for 30 s, 55°C annealing for 30 s and 72°C synthesis for 30 s. In addition, the house keeping gene L8 was amplified in parallel for comparison. In selected cases, real-time PCR was also performed for mRNA quantification.

### Analysis of DNA methylation

To analyze DNA methylation, DNA was first modified by bisulfite treatment according to the method described by Frommer et al. [34]. Modified DNA was amplified by PCR using primer pairs within the MHV-68 ORF K3 or its promoter region [35]. The primer sequences were as follows: BS: 5′-GATAAGGGGTAGATAGGTTTTT-3′ and 5′-ACTACCTCCTATTATCTAATAA-3′, BS1: 5′-TTTAGGTTGGGATTTTGTGGTT-3′ and 5′-AATTTTTAATACTACAAAATTC-3′, BS2: 5′-GATATTTTTTTTGAAGTATAAT-3′ and 5′-AAAATATAAACACAACTATATC-3′. The PCR conditions were as follows: initial denaturation at 95°C for 5 min, followed by 40 cycles of 95°C denaturation for 30 s, 45°C annealing for 40 s and 72°C synthesis for 60 s, and a final extension at 72°C for 10 min. After successful amplification, the PCR products were sequenced (Sequiserve, Vaterstetten, Germany).

### Statistical analysis

If not otherwise indicated, data were analyzed by Student's t-test.

## Results

### Generation and characterization of the 40 bp internal repeat and ORF M6 N-terminal mutants

**Figure 1:**
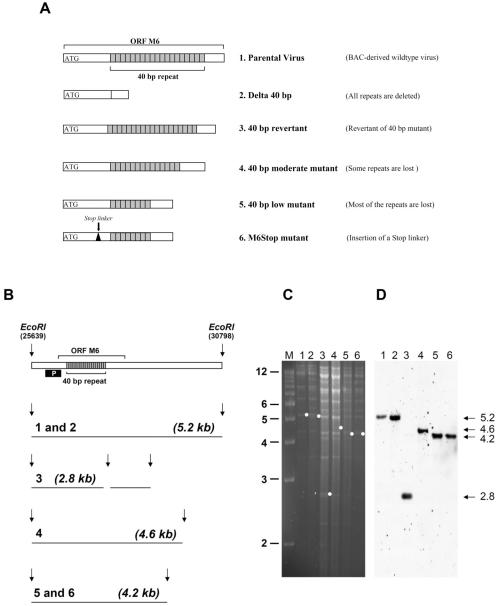
Generation of MHV-68 mutants. A) Schematic presentation of viral mutants. B) Scheme of the expected fragments after digestion of viral DNA with the restriction enzyme *EcoR*I. Digestion of DNA from both parental virus and the 40 bp revertant with *EcoR*I results in a 5.2 kb wildtype fragment. Deletion of the 40 bp repeat results in the loss of the 5.2 kb fragment and in the generation of a new 2.8 kb fragment. Partial loss of repeat units results in a shift of the 5.2 kb fragment to 4.6 kb and 4.2 kb fragments, respectively. “P” indicates the probe used for Southern blot analysis, corresponding to nucleotides 25889-26711. C) Structural analysis of reconstituted virus genomes by ethidium bromide-stained agarose gel analysis of viral DNA digested with *EcoR*I. Lane 1: Parental virus; Lane 2: 40 bp revertant; Lane 3: Delta 40 bp mutant; Lane 4: 40 bp moderate mutant; Lane 5: 40 bp low mutant; Lane 6: M6STOP mutant. D) Southern blot analysis of the gel shown in panel C using probe “P” indicated in panel B. The expected fragments are indicated by dots (panel C) or by arrows (panel D). Marker (M) sizes (in kilobase pairs) are indicated on the left.

To determine the role of the 40 bp internal repeat in pathogenesis, three mutants were constructed using MHV-68 cloned as bacterial artificial chromosome (BAC) (Fig. 1A). In the Delta 40 bp mutant, the repeat was completely deleted. Two additional clones were reconstituted to determine the significance of the number of repeat units. The 40 bp low mutant contains 15 repeat units and the 40 bp moderate mutant contains approximately 20 to 25 repeat units. To prove that any phenotype of the Delta 40 bp mutant is due to the deletion of the 40 bp repeat and not due to rearrangements outside the mutated region, a revertant virus was generated by replacing the 40 bp mutant fragment with a wild type genomic fragment. To determine a potential role of the hypothetical ORF M6, an N-terminal mutant of ORF M6 (M6STOP) was constructed (Fig. 1A). The BAC-cloned genomes were analyzed by restriction enzyme analysis with several restriction enzymes and in some cases by sequencing the mutated region (data not shown). The genomes of all reconstituted recombinant viruses were analyzed by restriction enzyme analysis (Fig. 1B and C) and Southern blot (Fig. 1D).

### Absence of the 40 bp internal repeat and ORF M6 does not significantly affect lytic replication *in vitro* and *in vivo*

**Figure 2:**
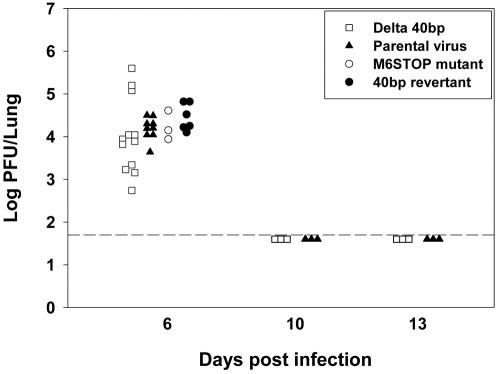
The 40 bp internal repeat is dispensable for lytic replication in the lung. C57BL/6 mice were i.n. infected with 5×10^4^ PFU. At the indicated time points after infection, lungs were harvested and the virus titers of lung homogenates were determined by plaque assay on BHK-21 cells. Each symbol represents a single mouse. The dashed line indicates the limit of detection which is 50 PFU [31].

To analyze the *in vitro* growth of the recombinant viruses, multi-step growth curves were performed on NIH3T3 cells. All mutant viruses replicated efficiently with similar kinetics and attained similar titers as both the parental and the 40 bp revertant virus (data not shown). To analyze lytic replication in the lungs of mice, C57BL/6 mice were intranasally (i.n.) infected and viral titers were determined in lung homogenates over time after infection. Overall, as *in vitro*, all mutant viruses replicated efficiently with similar kinetics and reached similar titers as the parental virus (Fig. 2). There was a trend towards a slightly reduced virus titer in lungs of mice infected with the Delta 40 bp mutant at day 6 after infection. The difference, however, was not statistically significant due to the variability between individual mice.

### The 40 bp internal repeat of MHV-68 is important for latency amplification

**Figure 3:**
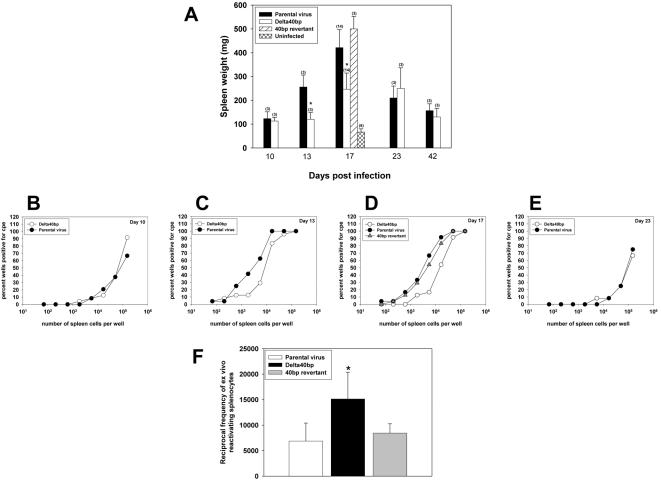
The 40 bp internal repeat is important for virus-induced splenomegaly and for *ex vivo* reactivation of latently infected splenocytes. C57BL/6 mice were i.n. infected with 5×10^4^ PFU. A) Extent of splenomegaly. At the indicated time points after infection, spleens were harvested and the spleen weight was determined. Shown are means±SD of the number of mice indicated in brackets. The asterisks indicate a statistically significant difference (p = 0.043 at day 13 and p = 1.02×10^−8^ at day 17; Student's t-test). The extent of *ex vivo* reactivation was determined 10 (B), 13 (C), 17 (D) and 23 (E) days after infection. Data shown are from a representative experiment with splenocytes pooled from 3 mice per group. F) Summary of independent *ex vivo* reactivation experiments performed with splenocytes at day 17 after infection. In each individual experiment, splenocytes from 3 mice per group were pooled. Shown are the reciprocal frequencies of reactivation (mean±SD; n = 4 for parental virus and Delta 40 bp mutant, n = 2 for revertant), calculated as described in materials and methods. The asterisk indicates a statistically significant difference (p = 0.040; Student's t-test).

To analyze the role of the 40 bp repeat during the latent phase of infection, C57BL/6 mice were i.n. infected and the extent of splenomegaly, the extent of *ex vivo* reactivation of latently infected splenocytes and the viral genomic load in the spleen were determined. At day 10 after infection, the spleen weight of C57BL/6 mice infected with the Delta 40 bp mutant was not significantly different when compared to the spleen weight of mice infected with the parental virus (Fig. 3A). However, at days 13 and 17 after infection, during the amplification phase of latency, the spleen weights of mice infected with the Delta 40 bp mutant virus were significantly lower than the spleen weights of mice infected with the parental virus. Importantly, at day 17 after infection, the spleen weight of mice infected with the revertant virus was not significantly different to the spleen weight of mice infected with the parental virus, clearly indicating that the observed phenotype of the Delta 40 bp mutant is due to the deletion of the 40 bp repeat and not to rearrangements outside of the mutated region. At days 23 and 42 after infection, the extent of splenomegaly caused by the Delta 40 bp mutant and the parental virus was again not significantly different.

**Figure 4:**
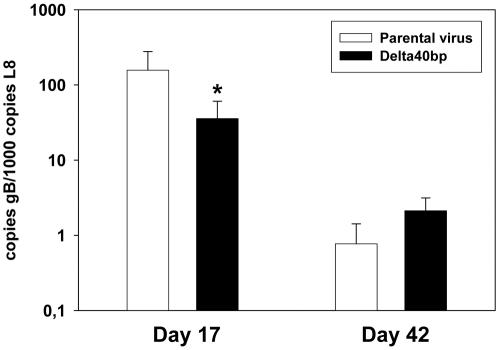
Viral genomic load in the spleen. Total DNA was extracted from spleens at days 17 and 42 after infection. Real time PCR was performed with 100 ng of total splenocyte DNA as described in materials and methods. Data shown are mean values from 3 to 4 individual mice, each tested in duplicate, compiled from two independent experiments. The asterisk indicates a statistically significant difference (p = 0.024; Student's t-test).

In addition to the spleen weight, we also determined the number of splenocytes reactivating in an *ex vivo* reactivation assay. At day 10 after infection, a similar number of splenocytes reactivated (Fig. 3B). However, at days 13 and 17 after infection, less splenocytes reactivated from mice infected with the Delta 40 bp mutant when compared to the parental virus (Fig. 3C and D), whereas the number of splenocytes reactivating from mice infected with the revertant virus was similar to the parental virus at day 17 after infection (Fig. 3D). A similar extent of reactivation was observed at day 23 after infection (Fig. 3E). The frequency of reactivating splenocytes at day 17 after infection was approximately 2-fold reduced after infection with the Delta 40 bp mutant (1 in 15135), when compared to the parental virus (1 in 6876) or to the revertant virus (1 in 8448) (Fig. 3F). Using real-time PCR, we determined the genomic load in spleens of mice infected with either the Delta 40 bp mutant or the parental virus. Consistent with the data from the *ex vivo* reactivation assay, the viral copy number from mice infected with the Delta 40 bp mutant (36 copies gB per 1000 copies L8) was approximately 4-fold lower at day 17 after infection, when compared to the parental virus (157 copies gB per 1000 copies L8) (Fig. 4). At day 42 after infection, the viral copy number detected after infection with both viruses was comparable. Since both measurements determine frequencies, they are not affected by the virus-driven splenomegaly which provides an indirect measure of viral colonization and which was also reduced when the 40 bp repeat was deleted (Fig. 3A). Thus, taken all three independent measurements of viral latency together, there was a substantial reduction in the amplification of latency after infection with MHV-68 lacking the 40 bp internal repeat.

### The ORF M6 of MHV-68 is not necessary for latency amplification

**Figure 5:**
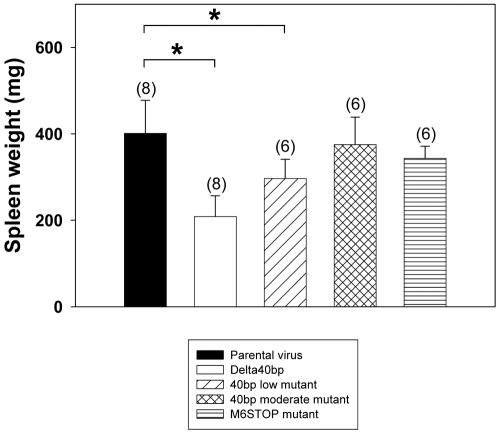
Splenomegaly is independent of ORF M6 expression. C57BL/6 mice were i.n. infected with 5×10^4^ PFU. At day 17 after infection, spleens were harvested and the spleen weight was determined. Shown are means±SD of the number of mice indicated in brackets. The asterisks indicate a statistically significant difference (p = 0.0001 for parental virus vs. Delta 40 bp mutant and p = 0.011 for parental virus vs. 40 bp low mutant; Student's t-test).

Although ORF M6 is unlikely to code for a protein [36], we nevertheless wanted to analyze a potential role of the hypothetical ORF M6 during the latent phase of infection. For this purpose, C57BL/6 mice were i.n. infected and both the extent of splenomegaly and the extent of *ex vivo* reactivation of latently infected splenocytes were determined. At day 17 after infection, the spleen weight of C57BL/6 mice infected with the M6STOP mutant was not significantly different when compared to the spleen weight of mice infected with the parental virus, whereas the number of 40 bp repeat units correlated with the spleen weight (Fig. 5). In addition, the extent of *ex vivo* reactivation was similar from splenocytes of mice infected with the M6STOP mutant when compared to the parental virus (data not shown).

### The absence of CD8^+ ^T cells partially reverses the phenotype of the Delta 40 bp mutant

**Figure 6:**
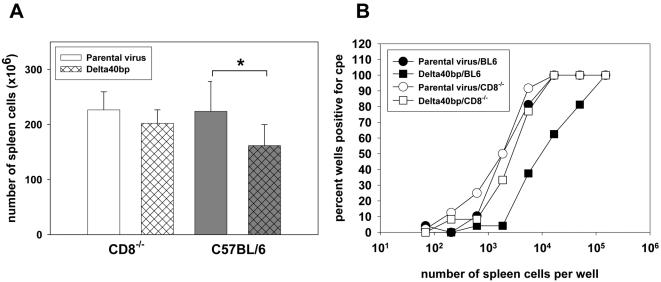
The absence of CD8^+ ^T cells partially reverses the phenotype of the Delta 40 bp mutant. A) Splenomegaly. C57BL/6 or CD8^−/−^ mice (5 mice per group) were i.n. infected with 5×10^4^ PFU. At day 17 after infection, spleens were harvested and the number of spleen cells was determined. Data shown are means±SD. The asterisk indicates a statistically significant difference (p = 0.01; Student's t-test). B) *Ex vivo* reactivation. C57BL/6 mice or CD8^−/−^ mice were i.n. infected with 5×10^4^ PFU. The extent of *ex vivo* reactivation was determined 17 days after infection. Splenocytes pooled from 5 mice per group were used.

The phenotype of the Delta 40 bp mutant resembled to some extent the phenotype of the ORF K3 mutant of MHV-68 [37]. K3 is involved in evasion from CD8^+^ T cell immunity. Therefore, we tested the hypothesis that the 40 bp repeat might also be involved in evasion from the CD8^+^ T cell immune response. For this purpose, CD8^−/− ^mice and as a control, C57BL/6 mice, were infected i.n., and both the extent of splenomegaly and *ex vivo* reactivation were determined 17 days after infection. In contrast to C57BL/6 mice, the spleen cell numbers of CD8^−/− ^mice infected either with the parental virus or with the Delta 40 bp mutant were not significantly different (Fig. 6A). In addition, the extent of *ex vivo* reactivation of splenocytes isolated from CD8^−/− ^mice infected either with the parental virus or with the Delta 40 bp mutant was, in contrast to splenocytes from C57BL/6 mice, similar (Fig. 6B). Similar results were obtained after infection of beta-2-microglobulin^−/−^ mice which also lack CD8^+^ T cells [38] (data not shown).

### The 40 bp internal repeat of MHV-68 is involved in latency amplification by regulating the expression of latency-associated genes

**Figure 7:**
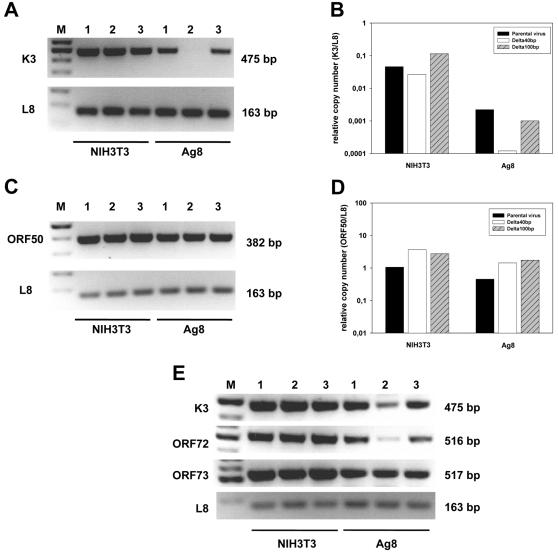
The 40 bp internal repeat of MHV-68 is involved in latency amplification by regulating the expression of latency-associated genes. A) RT-PCR analysis of the expression of K3 after infection of fibroblasts (NIH3T3) and B cells (Ag8). As control, the expression of the murine ribosomal protein L8 gene, which was amplified in parallel, was determined. Lanes 1: Parental virus; Lanes 2: Delta 40 bp mutant; Lanes 3: Delta 100 bp mutant; M: marker. The sizes of the PCR products are indicated on the right. B) Quantitative RT-PCR analysis of the expression of K3 after infection of fibroblasts (NIH3T3) and B cells (Ag8). The data are presented as relative copy number of K3 to L8. C) Determination of the viral genomic load by PCR using primers specific for ORF 50 of MHV-68. As control, the murine ribosomal protein L8 gene was amplified in parallel. Lanes 1: Parental virus; Lanes 2: Delta 40 bp mutant; Lanes 3: Delta 100 bp mutant; M: marker. The sizes of the PCR products are indicated on the right. D) Quantitative PCR analysis of the genomic load after infection of fibroblasts (NIH3T3) and B cells (Ag8). The data are presented as relative copy number of ORF50 to L8. E) RT-PCR analysis of the expression of K3, ORF72 and ORF73 after infection of fibroblasts (NIH3T3) and B cells (Ag8). As control, the expression of the murine ribosomal protein L8 gene, which was amplified in parallel, was determined. Lanes 1: Parental virus; Lanes 2: Delta 40 bp mutant; Lanes 3: 40 bp revertant; M: marker. The sizes of the PCR products are indicated on the right. The data shown in figure 7 are from representative experiments which were repeated 3 times with similar results.

To find out whether the 40 bp internal repeat of MHV-68 is involved in the amplification of latency by regulating the expression of viral genes, we analyzed the expression of K3, a gene which has been shown to be expressed both during lytic and latent infection [37]. First, we analyzed the expression of K3 during lytic replication by infection of NIH3T3 cells with parental virus, the Delta 40 bp mutant or, as a control, with a mutant lacking the 100 bp repeat (H. Adler et al.; unpublished). Expression of K3 was tested by RT-PCR. In NIH3T3 cells, the expression of K3 showed no differences (Fig. 7A, B and E). We then analyzed the expression of K3 during latent infection using the murine B cell line Ag8 as a model. To demonstrate that MHV-68 establishes a latent infection in this cell line, Ag8 cells were infected with MHV-68 (MOI of 1) for 24 hr and then washed thoroughly with medium. Infected cells were then used in the reactivation assay which measures viral reactivation in culture. As has been shown previously for infection of naive mouse splenocytes [39], reactivation required intact viable cells since cultures using disrupted cells barely showed any cytopathic effect (cpe) indicating that the majority of reactions observed in the assay were due to viral reactivation from latency (data not shown). In contrast to NIH3T3 cells, the expression of K3 was strongly reduced in Ag8 cells (Fig. 7A, B and E). The reduction of K3 expression in Ag8 cells was specifically observed after infection with the Delta 40 bp mutant since it was not seen after infection with a mutant lacking the 100 bp repeat. Furthermore, the reduction was not due to a less efficient infection of Ag8 cells by the Delta 40 bp mutant since the viral genomic load, as determined by quantitative PCR, was similar in all samples analyzed (Fig. 7C and D). To further evaluate a potential role of the 40 bp repeat in regulation of expression of latency-associated genes, we additionally analyzed the expression of ORF72 and ORF73, selected genes which are located more than 70 kbp away from the 40 bp repeat and which have also been shown to be expressed both during lytic and latent infection [40]–[43]. As for K3, both ORF72 and ORF73 were expressed to a similar extent in NIH3T3 cells after infection with the parental virus, the Delta 40 bp mutant and the revertant. In contrast, the expression of ORF72 was reduced in Ag8 cells after infection with the Delta 40 bp mutant, whereas the expression of ORF73 was not reduced (Fig. 7E). Importantly, the expression levels after infection with the revertant virus were very close, although not completely identical, to those of the parental virus. The small reduction is most likely due to a slightly lower number of repeat units present in the revertant virus when compared to the parental virus (see Fig. 1B).

### The promoter region of K3 is not methylated after infection of B cells with the Delta 40 bp mutant

To test the hypothesis whether the reduced expression of K3 after infection of Ag8 cells with the Delta 40 bp mutant might be related to differences in the methylation status of the gene, we decided to assay methylation of the promoter region of K3. The chemical modification of cytosine to uracil by bisulfite treatment provides a method to study DNA methylation. In this reaction, all cytosines are converted to uracil, but those that are methylated (5-methylcytosine) are resistant to this modification. The altered DNA can then be amplified and sequenced, providing detailed information within the amplified region of the methylation status of all CpG sites [34]. Thus, we isolated DNA from Ag8 cells after infection with the Delta 40 bp mutant or the parental virus and subjected it to bisulfite treatment. Modified DNA was then amplified by PCR and the PCR products were sequenced. There was complete conversion of all cytosines to uracil in both sequences, indicating that they were not methylated and that there was no difference in the methylation status within the analyzed region (data not shown).

## Discussion

The genomes of gammaherpesviruses contain variable numbers of internal repeats whose precise role for *in vivo* pathogenesis is not well understood. In this study, we investigated the biological role of the 40 bp internal repeat of MHV-68 by infecting mice with mutant viruses partially or completely lacking this repeat. The 40 bp internal repeat appears to be largely dispensable for lytic replication both *in vitro* and *in vivo*, however, it seems to play an important role during the amplification of latency. This phenotype seems to be dependent on the number of the 40 bp repeat units present in the genome and is not associated with the expression of the hypothetical ORF M6. The latter is consistent with a recent report which considers ORF M6 unlikely to code for a protein [36]. The expression of the latency-associated genes K3 and ORF72 but not ORF73 is reduced after infection of the murine B cell line Ag8, but not after infection of NIH3T3 fibroblasts, with a mutant virus lacking the 40 bp repeat. Future studies will aim to analyze whether the observed differences in viral gene expression reflect cell type-specific differences or are just reflecting properties of the cell lines used as models in this study. Nevertheless, the data are suggestive for a role of the 40 bp repeat in latency amplification and regulation of viral gene expression.

Latency of herpesviruses is accompanied by silencing of most of their genes present in the genome. However, during latency, herpesviruses express a subset of specific genes which play an important role in immune evasion and maintenance of episomal DNA [44]. For MHV-68, it has been shown that genes from three distinct regions of the genome are transcriptionally active during the latent phase of infection [33], [45], [46]. How transcription of these genes is regulated is largely unknown. Our data suggest that the 40 bp internal repeat of MHV-68 plays an important role in the regulation of the expression of some but not all latency-associated genes since the expression of two out of three selected genes distributed across the genome was markedly reduced in Ag8 B cells but not in NIH3T3 fibroblasts in the absence of the 40 bp repeat. Importantly, the observed phenotype was not dependent on the expression of the hypothetical ORF M6 but seemed to be a function of the 40 bp repeat per se. In addition, the reduction in expression of the latency-associated genes was specific for the 40 bp repeat since it was not observed in the absence of the 100 bp repeat. The consequence for the pathogenesis was a significant reduction in latency amplification, reflected by a reduced extent of splenomegaly, a lower number of reactivating splenocytes and a reduced genomic load during the amplification phase. To achieve the burst of lymphocyte proliferation which initially establishes the latent pool, gammaherpesviruses have to escape the host immune control [47]. The K3 gene of MHV-68 has been shown to play an important role in CD8^+^ T cell immune evasion [48] by degrading MHC class I heavy chains [49] and TAP [50]. Disruption of K3 resulted in a reduction of latency amplification [37]. We also observed, albeit to a lesser extent, a reduction of latency amplification after deletion of the 40 bp internal repeat. The defect in the amplification of latency after disruption of K3 was completely reversible by CD8^+^ T cell depletion [37]. In our study, the absence of CD8^+^ T cells in CD8-deficient mice partially reversed the phenotype of the Delta 40 bp mutant. Besides K3, also ORF72 expression was reduced when the 40 bp repeat was deleted, providing a possible explanation why the reversion was not complete. K3 is in close proximity to the 40 bp repeat, ORF72, however, is rather distal. Thus, the expression of additional latency-associated or other viral genes whose expression we did not test might also be affected by the absence of the 40 bp repeat. However, not all genes are affected since the expression of ORF73 was not reduced in the absence of the 40 bp repeat. ORF73 is the MHV-68 episome maintenance protein and its transcription is driven by three different promoters, two of them are in the viral terminal repeats [51].

EBV selectively methylates and silences regions of its genome which would, in the infected host, be detrimental to viral persistence, whereas other viral genes, which are critical for propagation of viral DNA, are expressed from alternative promoters which are protected from the host cell methylation machinery [52]. We exemplarily assayed the methylation status of the promoter region of K3 in the wildtype and Delta 40 bp repeat mutant, but we could not detect differences in the methylation status within the analyzed region. Yet, the 40 bp repeat might contribute to other epigenetic regulation mechanisms such as histone acetylation or through a direct role in chromatin structure thus influencing gene transcription [53]. In eukaryotic cells, active and inactive genes reside adjacent to one another. Motifs such as silencers, enhancers and locus control regions (LCRs) act over long distances to regulate adjacent genes, and so called insulators prevent the misregulation of adjacent genes by restricting the effects of regulatory elements to specific regions [54], [55]. LCRs are sets of elements that are sufficient to activate linked genes in a tissue-specific, copy-number-dependent manner [56]. Similarly, active and inactive viral genes reside adjacent to one another and must be tightly regulated during herpesvirus latency. Thus, we propose that the 40 bp repeat might function as a regulatory element. The mechanisms by which direct repeats may influence gene regulation are not completely understood but the formation of nonstandard DNA conformations, for example looping, which could either directly interfere with transcription or could be recognized by factors only present in particular cell types, have been proposed [54], [57], [58]. Interestingly, by computer prediction, the 40 bp repeat seems to be able to form strong secondary structures.

Defects in latent gene expression and impaired immune evasion might explain the phenotype of the Delta 40 bp repeat mutant, yet there are also other possibilities: First, we have recently demonstrated that the 40 bp repeat forms part of one lytic origin of MHV-68 [59]. Thus, although having no effect *in vitro*, deletion of the 40 bp repeat might lead to impairment of viral reactivation *in vivo*. How herpesvirus lytic origins are activated in an authentic environment *in vivo* is not completely understood. It could be possible that the second lytic origin at the right end of the genome prevents the obvious manifestation of any defect in *in vitro* assays, yet is unable to completely complement the left origin *in vivo*. We indeed observed a slightly reduced virus titer in lungs of mice infected with the Delta 40 bp mutant. Second, since inflammation is one of the factors which might play an important role in reactivation *in vivo*, altered inflammatory signals might also contribute to the partial rescue seen in CD8-deficient mice. The precise role of the 40 bp repeat in molecular terms thus remains elusive and deserves further studies.

As described previously, the 40 bp repeat has the propensity to be unstable in *E. coli* [24], [25]. As a result, some of the 40 bp repeat units are almost always lost during the generation of mutants using MHV-68 cloned as a BAC. Our study demonstrated that only the complete deletion of all 40 bp repeat units, but not the loss of some, resulted in an obvious phenotype *in vivo*. Thus, our results further corroborate the usefulness of the MHV-68 BAC for the fast and efficient generation of mutants.

In summary, our data suggest that the 40 bp repeat is involved in latency amplification and regulation of viral gene expression. We show for the first time a role for a gammaherpesviral internal repeat in the pathogenesis. Future investigations will aim to analyze in detail the molecular mechanisms underlying the regulatory function of the 40 bp repeat in terms of transcription of viral genes.
